# Effect of Indoor air pollution from biomass and solid fuel combustion on symptoms of preeclampsia/eclampsia in Indian women

**DOI:** 10.1111/ina.12144

**Published:** 2014-08-11

**Authors:** S Agrawal, S Yamamoto

**Affiliations:** 1 South Asia Network for Chronic Disease, Public Health Foundation of IndiaNew Delhi, India; 2 Department of Non-Communicable Disease Epidemiology, London School of Hygiene and Tropical MedicineLondon, UK

**Keywords:** Preeclampsia/eclampsia, Biomass fuels, Solid fuels, Cooking smoke, Women, Indoor air pollution, NFHS-3, India

## Abstract

Available evidence concerning the association between indoor air pollution (IAP) from biomass and solid fuel combustion and preeclampsia/eclampsia is not available in developing countries. We investigated the association between exposure to IAP from biomass and solid fuel combustion and symptoms of preeclampsia/eclampsia in Indian women by analyzing cross-sectional data from India's third National Family Health Survey (NFHS-3, 2005–2006). Self-reported symptoms of preeclampsia/eclampsia during pregnancy such as convulsions (not from fever), swelling of legs, body or face, excessive fatigue or vision difficulty during daylight, were obtained from 39 657 women aged 15–49 years who had a live birth in the previous 5 years. Effects of exposure to cooking smoke, ascertained by type of fuel used for cooking on preeclampsia/eclampsia risk, were estimated using logistic regression after adjusting for various confounders. Results indicate that women living in households using biomass and solid fuels have two times higher likelihood of reporting preeclampsia/eclampsia symptoms than do those living in households using cleaner fuels (OR = 2.21; 95%: 1.26–3.87; *P* = 0.006), even after controlling for the effects of a number of potentially confounding factors. This study is the first to empirically estimate the associations of IAP from biomass and solid fuel combustion and reported symptoms suggestive of preeclampsia/eclampsia in a large nationally representative sample of Indian women and we observed increased risk. These findings have important program and policy implications for countries such as India, where large proportions of the population rely on polluting biomass fuels for cooking and space heating. More epidemiological research with detailed exposure assessments and clinical measures of preeclampsia/eclampsia is needed in a developing country setting to validate these findings.

**Practical Implications**

The findings from this study have important program and policy implications for countries such as India, where large proportions of the population rely on polluting biomass fuels for cooking and space heating, and have an additional burden of high maternal and infant mortality and morbidity, and preeclampsia/eclampsia is one of a major cause for this high burden. With the target of the Millennium Development Goals in sight, preeclampsia/eclampsia needs to be identified as a priority area in reducing maternal mortality and morbidity in developing countries including India.

## Introduction

Preeclampsia is a pregnancy-induced hypertensive disorder characterized by high blood pressure and proteinuria after the 20th week of pregnancy (Sibai et al., 2005). Eclampsia is defined as the occurrence of generalized seizures/convulsions and/or unexplained coma during pregnancy or postpartum in a preeclamptic woman in the absence of other neurologic conditions (such as epilepsy) (Aagaard-Tillery and Belfort, 2005; Sibai, 2005). Preeclampsia/eclampsia is thus a potentially fatal disorder in pregnant women and remains one of the leading causes of maternal mortality and morbidity worldwide and is associated with adverse pregnancy outcomes including perinatal death, preterm birth, and intrauterine growth retardation (Sibai et al., 2005). Established risk factors for preeclampsia/eclampsia reported in high-income country settings include young and old maternal age, obesity prior to pregnancy, being unmarried, excessive weight gain during pregnancy, multiple gestation, nulliparity, chronic hypertension, low socioeconomic status, prolonged birth interval, lack of prenatal care, and current smoking (Ansari et al., 1995; Baeten et al., 2001; Chesley, 1984; Coghill et al., 2011; Douglas and Redman, 1994; Saftlas et al., 1990; Zwart et al., 2008).

An emerging risk factor for preeclampsia/eclampsia, particularly in low- and middle-income countries (LMICs), is the indoor air pollution. Indoor air pollution, from traditional fuels (such as biomass and coal) and cooking stoves, is associated with an increase in the incidence of respiratory infections, including pneumonia, tuberculosis and chronic obstructive pulmonary disease, low birthweight, cataracts, cardiovascular events, and all-cause mortality both in adults and children (Bruce et al., 2000; Fullerton et al., 2008; WHO, 2014). According to WHO (2014), an estimated 4.3 million people a year die prematurely from illness attributable to the household air pollution caused by the inefficient use of solid fuels (from 2012 data). Among these deaths, 12% are due to pneumonia, 34% from stroke, 26% from ischemic heart disease, 22% from chronic obstructive pulmonary disease (COPD), and 6% from lung cancer. The poorest and most vulnerable populations in developing countries are generally the most exposed to indoor air pollution from biomass combustion (Mishra, 2003; Agrawal, 2012; Smith et al., 2013). Exposure levels are usually much higher among women as they do most of the cooking (Behera et al., 1988) and among young children because they are often carried or held on their mother's back or lap during cooking times (Albalak, 1997). Recent studies in India (Epstein et al., 2013; Lakshmi et al., 2013) suggest that household use of coal and kerosene was also associated with an increased risk of low birthweight, still births, and neonatal deaths.

Over 200 studies that have measured air pollution levels in developing country households across all WHO regions (The Global Indoor Air Pollution Database[Bibr b75]), provide clear evidence of extreme exposures in solid cooking fuel using settings, often manifold higher than recommended WHO Air Quality Guidelines (AQGs) (Bonjour et al., 2013; World Health Organization Regional Office for Europe, 2005). The burning of solid fuels indoors in open fires or traditional cooking stoves (*chulhas*) results in high levels of toxic pollutants in the kitchen area (Duflo et al., 2008; Smith et al., 2000, 2004, 2012). Poor ventilation likely worsens the adverse health effects of indoor air pollution (Reddy et al., 2004). Findings from India's third National Family Health Survey (NFHS-3, 2005–2006) showed that 90% of rural and 32% of urban households (overall 71%) in India use biomass and solid cooking fuels that generate smoke and unhealthy conditions when inhaled (IIPS & Macro International, 2007). Additionally, 74% of households cook their meals in the house. About one-third of households that cook inside the house do not have a separate room for cooking. In both urban and rural areas, 9 in 10 households that use solid fuels and cook on an open fire also lack chimneys to divert smoke (IIPS & Macro International, 2007). Smith (1999) reported between 400 000 and 550 '000 premature deaths annually among adult women and children aged <5 years in India arising from exposure to indoor air pollution. Using a disability-adjusted lost life-year approach, the total is 4–6% of the Indian national burden of disease, placing indoor air pollution as a major risk factor in the country (Smith, 2000). It has been estimated that, exposure to indoor air pollution may be responsible for nearly 2 million excess deaths in developing countries and for some 4% of the global burden of disease (Bruce et al., 2000).

In India, biomass and solid fuels are typically burned indoors in simple household cook stoves, such as a pit, three pieces of brick, or a U-shaped construction made from mud, which burn these fuels inefficiently and do not often include flues or hoods. Under these conditions, high levels of health-damaging airborne pollutants, including PM10, CO, NOx, SOx (more from coal), formaldehyde, and dozens of toxic polycyclic aromatic hydrocarbons (e.g. benzo[a]pyrene) and other organic matter, can be generated indoors. The individual peak and mean exposures experienced in such settings are often much greater than guideline levels recommended by the World Health Organization (WHO, 1997; World Health Organization Regional Office for Europe, 2005; Bonjour et al., 2013). A recent study by Balakrishnan et al. (2013) reported that the measured mean 24-h concentration of PM2.5 in solid cook fuel-using households ranged from 163 *μ*g/m^3^ (95% CI: 143–183; median 106; IQR: 191) in the living area to 609 *μ*g/m^3^ (95% CI: 547–671; median: 472; IQR: 734) in the kitchen area. After extrapolating the household results by state to all solid cooking fuel-using households in India covered by the NFHS 2005–2006, the same study found an estimate of 450 *μ*g/m^3^ (95% CI: 318–640) and 113 *μ*g/m^3^ (95% CI: 102–127) for national average 24-h PM2.5 concentrations in the kitchen and living areas, respectively (Balakrishnan et al., 2013).

Previous findings regarding the association between air pollution and preeclampsia/eclampsia, mostly conducted in developed countries, are limited and have been inconsistent (Lee et al., 2013; Pereira et al., 2013; Rudra et al., 2011; Wu et al., 2009). Several recent studies have reported positive associations between preeclampsia and air pollutants including nitrogen oxides (NOx), nitrogen monoxide (NO), nitrogen dioxide (NO2), carbon monoxide (CO), ozone (O3), particulate matter <2.5 *μ*m in aerodynamic diameter (PM2.5), and particulate matter <10 *μ*m in aerodynamic diameter (PM10) (Lee et al., 2012; Wu et al., 2009, 2011), but others have reported no association with PM2.5 or CO (Rudra et al., 2011) or inconclusive findings for PM10 and NO2 (van den Hooven et al., 2011). Two studies have reported positive associations between gestational hypertension (a risk factor and early symptom of preeclampsia) and air pollutants [PM10 and PM2.5 (Vinikoor-Imler et al., 2012) and NO2 and PM10 (van den Hooven et al., 2011)]. A study in Spain also observed an increased risk of preeclampsia associated with exposure to fine particulate air pollution (Dadvand et al., 2013).

It is seen from the above discussion that biomass and solid fuels are a major source of indoor air pollution, but in low- and middle-income countries such as India, the adverse health effects, including preeclampsia/eclampsia, of exposure among pregnant women are poorly understood. To our knowledge, very few/no epidemiological studies have examined associations between indoor air pollution and preeclampsia/eclampsia among Indian women. In the present study, we aimed to examine the effect of exposure to cooking smoke from biomass and solid fuel combustion on the risk of preeclampsia/eclampsia using data from a large-scale cross-sectional nationally representative sample of adult women in India.

## Methods

### Data

Cross-sectional data from India's third National Family Health Survey (NFHS-3), conducted during 2005–2006, were used for this analysis. NFHS was designed along the lines of the Demographic and Health Surveys (available at www.measuredhs.com) that have been conducted in many LMICs since the 1980s. The NFHS has been conducted in India for three successive rounds, each at an interval of 5 years. The most recent NFHS, that is NFHS-3, collected demographic, socioeconomic and health information from a nationally representative probability sample of 124 385 women aged 15–49 years residing in 109 041 households. The sample is a multistage cluster sample with an overall response rate of 98%. All states of India are represented in the sample (except the small Union Territories), covering more than 99% of the country's population. Full details of the survey have been published (IIPS & Macro International, 2007) and also available at www.nfhsindia.org. The analysis presented in this study focuses on 39 657 women from the total sample who reported being ever married and who had a live birth in the 5 years preceding the survey.

### Response variable

NFHS-3 included several questions related to health problems of women during pregnancy for the most recent live birth in the 5 years preceding the survey. Mothers were asked if at any time during their last pregnancy they experienced symptoms of convulsions (not from fever), swelling of the legs, body or face, excessive fatigue, have difficulty with vision during daylight, or vaginal bleeding. According to the WHO criteria for assessing the occurrence of preeclampsia symptoms, women who reported difficulty with vision during daylight, and swelling of the legs, body, or face were coded as having symptoms of preeclampsia (WHO, 2003a), whereas those who reported experiencing convulsions (not from fever) were coded as symptomatic of eclampsia (WHO, 2003a). We used this as a proxy measure for preeclampsia/eclampsia. However, it was not possible to confirm clinical diagnosis of these symptoms. Data on blood pressure and proteinuria during pregnancy, which are typical clinical diagnostic markers of preeclampsia (Roberts et al., 2003), were not available in the NFHS-3. Data on physician reported diagnosis of convulsions/seizures were also not available in the NFHS-3 to verify a self-reported diagnosis.

### Predictor variable

Exposure to cooking smoke was determined indirectly by the type of fuel used for cooking in the household. The survey used a 10-item classification of cooking fuel: electricity, liquefied petroleum gas (LPG)/natural gas, biogas, kerosene, coal/lignite, charcoal, wood, straw/shrubs/grass, agricultural crop waste, dung cakes, and a residual category of other fuels (unknown). The question asked was, ‘What type of fuel does your household mainly use for cooking?’, followed by the above list of fuels. We used information from the above questions to group households into two categories representing the extent of exposure to cooking smoke: high- and medium-exposure group (households using either biomass fuels—such as, wood, straw/shrubs/grass, agricultural crop waste, dung cakes, or others—or solid fuels—such as coal/lignite and charcoal); and low-exposure group (households using only cleaner fuels—such as kerosene, LPG/natural gas, biogas, or electricity).

### Covariates/confounders

Preeclampsia/eclampsia is a complex disease, likely to be influenced by several other factors, so we included potential covariates and confounders in the models which were selected on the basis of previous knowledge of their association with preeclampsia/eclampsia. The following maternal reproductive risk factors were evaluated: parity (1, 2–3, 4+); type of pregnancy (singleton, twin); history of a terminated pregnancy (no, yes). Health- and lifestyle-related factors included body mass index (BMI) kg/m^2^ categories (Indian adult population standard) (Indian Consensus Group, 1996): ≤18.4 kg/m^2^ (underweight), 18.5–22.9 kg/m^2^ (normal), 23.0–24.9 kg/m^2^ (overweight), ≥25 kg/m^2^ (obese); current tobacco smoking (no, yes); alcohol use (no, yes); self-reported diabetes (no, yes); self-reported asthma (no, yes); anemia level (not anemic, mild, moderate, severe). Studies have shown that women with preeclampsia had a higher BMI, and higher systolic and diastolic blood pressure (Kaaja et al., 2005), but a protective effect of prenatal alcohol consumption with preeclampsia was found in some studies (Salihu et al., 2011; Kiondo et al., 2012; McCarthy et al., 2013). Anemia during pregnancy is a major health problem, and some studies found that the greater the severity of the anemia during pregnancy, the greater was the risk of preeclampsia (Ali et al., 2011). Some studies also suggest that asthmatics, particularly those who are symptomatic during pregnancy, may be at a higher risk of developing preeclampsia (Rudra et al., 2006). Socio-demographic predictors included age (15–29, 30–39, 40–49 years); education (no education, primary, secondary, higher); religion (Hindu, Muslim, Christian, Sikhs, others); caste (scheduled castes, scheduled tribes, other backward class, others); wealth index (measured by an index based on household ownership of assets and graded as lowest, second, middle, fourth, and highest) which was computed using previously described methods (see Data S1 for the advantage and disadvantage of using the wealth index and items used to compute wealth index in NFHS-3); place of residence (urban, rural); and geographic regions (north, northeast, central, east, west, south). For definition of selected variables see, Table1. Table S1 gives the descriptive statistics of the covariates and the main predictor variable, that is, household cooking fuel use.

**Table 1:** Sample distribution and reported unadjusted prevalence of preeclampsia/eclampsia during pregnancy for the most recent live birth in the 5 years preceding the survey among women aged 15–49 years (*n* = 39 657) according to household cooking fuel use and other selected characteristics, India, 2005–2006

Characteristics	Sample distribution		Preeclampsia/Eclampsia		*χ*^2^ *P* value
	Number	Percent	Number	Percent	
Household cooking fuel use^a^					
Clean fuel	7969	22.1	34	0.4	<0.0001
Biomass and solid fuel	28 158	77.9	397	1.4	
Maternal factors					
Parity					
1	10 453	26.4	85	0.8	<0.0001
2–3	18 199	45.9	184	1.0	
4+	11 005	27.8	187	1.7	
Type of pregnancy					
Singleton	39 298	99.1	446	1.1	0.003
Twin	359	0.9	11	3.1	
Terminated pregnancy^b^					
No	32 319	81.5	350	1.1	0.005
Yes	7338	18.5	106	1.4	
Health and lifestyle factors					
Body mass index^c^					
Underweight (≤18.5 kg/m^2^)	11 592	30.5	183	1.3	0.001
Normal (18.5–22.9 kg/m^2^)	20 714	54.4	224	1.3	
Overweight (23.0–24.9 kg/m^2^)	2770	7.3	23	0.8	
Obese (≥25.0 kg/m^2^)	3226	14.7	15	0.5	
Current tobacco smoking					
No	39 049	98.5	446	1.1	0.164
Yes	608	1.5	10	1.7	
Drinks alcohol					
No	38 735	97.7	433	1.1	<0.0001
Yes	911	2.3	24	2.6	
Diabetes^d^					
No	39 123	98.7	448	1.1	0.240
Yes	160	1.3	8	1.6	
Asthma^d^					
No	39 163	98.8	434	1.1	<0.0001
Yes	470	1.2	22	4.7	
Anemia level^e^					
Not anemic	14 939	40.1	129	0.9	<0.0001
Mild	15 082	40.4	200	1.3	
Moderate	6616	17.7	101	1.5	
Severe	652	1.7	6	0.9	
Background factors					
Age					
15–29	29 190	73.6	309	1.1	0.013
30–39	9421	23.8	134	1.4	
40–49	1047	2.6	14	1.3	
Education^f^					
No education	18 783	47.4	294	1.6	<0.0001
Primary	5550	14.0	66	1.2	
Secondary	12 959	32.7	91	0.7	
Higher	2365	6.0	5	0.2	
Religion					
Hindu	31 280	78.9	362	1.2	0.027
Muslim	6482	16.3	69	1.1	
Christian	814	2.1	6	0.7	
Sikhs	514	1.3	4	0.8	
Others^g^	568	1.4	14	2.5	
Caste/tribe^h^					
Scheduled caste	7945	20.1	99	1.2	<0.0001
Scheduled tribes	3742	9.5	84	2.2	
Other backward class	15 878	40.2	173	1.1	
General	10 845	27.5	88	0.8	
Missing caste	1089	2.8	11	1.0	
Wealth index^i^					
Lowest	9566	24.1	187	2.0	<0.0001
Second	8600	21.7	109	1.3	
Middle	7769	19.6	76	1.0	
Fourth	7256	18.3	47	0.6	
Highest	6466	16.3	36	0.6	
Place of residence					
Urban	10 622	26.8	65	0.6	<0.0001
Rural	29 035	73.2	392	1.4	
Geographic regions^j^					
North	5678	12.8	52	1.0	<0.0001
Northeast	1613	4.1	22	1.4	
Central	11 111	28.0	188	1.7	
East	10 042	25.3	137	1.4	
West	5117	12.9	36	0.7	
South	6696	16.9	21	0.3	
India Total^k^	39 657		456	1.2	

a Clean fuels include kerosene, liquefied petroleum gas/natural gas, biogas, or electricity; biomass fuels include wood, straw/shrubs/grass, agricultural crop waste, dung cakes, others; and solid fuels include such as coal/lignite or charcoal.

b Includes both miscarriages/spontaneous abortion and induced abortion.

c In NFHS-3, all respondents were weighed using a solar-powered scale with an accuracy of ± 100 g. Their height was measured using an adjustable wooden measuring board, specifically designed to provide accurate measurements (to the nearest 0.1 cm). Women who were pregnant at the time of the survey, or who had given birth during the 2 months preceding the survey, were excluded from these anthropometric measurements.

d From self-reports only.

e Mild anemia (Hemoglobin 10.0–10.9 g/dl for pregnant women, 10.0–11.9 g/dl for non-pregnant women, and 12.0–12.9 g/dl for men), moderate anemia (7.0–9.9 g/dl for women and 9.0–11.9 g/dl for men), and severe anemia (<7.0 g/dl for women and <9.0 g/dl for men). In the survey, appropriate adjustments in these cutoff points were made for respondents living at altitudes above 1000 m and respondents who smoke, as both of these groups require more hemoglobin in their blood (Centers for Disease Control and Prevention, 1998).

f Education: No education (0 years of education), primary (1–5 years of education), secondary (6–8 years of education), higher (9+ years of education).

g Others include Sikh, Buddhist, Christian, Jain, Jewish, Zoroastrian.

h Scheduled *c*astes and scheduled tribes are identified by the Government of India as socially and economically backward and needing protection from social injustice and exploitation. Other backward class is a diverse collection of intermediate castes that were considered low in the traditional caste hierarchy but are clearly above scheduled castes. Others are thus a default residual group that enjoys higher status in the caste hierarchy.

i Items comprising the wealth index in the third National Family Health Survey household includes electrification; type of windows; drinking water source; type of toilet facility; type of flooring; material of exterior walls; type of roofing; cooking fuel; house ownership; number of household members per sleeping room; ownership of a bank or post office account; and ownership of a mattress, a pressure cooker, a chair, a cot/bed, a table, an electric fan, a radio/transistor, a black and white television, a color television, a sewing machine, a mobile telephone, any other telephone, a computer, a refrigerator, a watch or clock, a bicycle, a motorcycle or scooter, an animal-drawn cart, a car, a water pump, a thresher and a tractor.

j Region: North: Delhi, Haryana, Himachal Pradesh, Jammu and Kashmir, Punjab, Rajasthan, Uttaranchal; Northeast: Assam, Arunachal Pradesh, Manipur, Meghalaya, Mizoram, Nagaland, Sikkim, Tripura; Central: Chhattisgarh, Madhya Pradesh, Uttar Pradesh; East: Bihar, Jharkhand, West Bengal, Orissa; West: Maharashtra, Goa, Gujarat; South: Andhra Pradesh, Karnataka, Kerala, Tamil Nadu.

k Number of women varies slightly for individual variables depending on the number of missing values.

### Statistical analysis

Because our response variable—preeclampsia/eclampsia—is dichotomous, we used logistic regression to estimate the effects of cooking smoke (from biomass and solid fuel use relative to cleaner fuel use) with seven socioeconomic and demographic variables, three maternal factors, and six health- and lifestyle-related factors mentioned above. Results are presented as odds ratios with 95% confidence intervals (OR with 95% CI). The estimation of confidence intervals takes into account design effects due to clustering at the level of the primary sampling unit. We assessed the possibility of multicollinearity between the covariates. In the correlation matrix of covariates, all pairwise Pearson's correlation coefficients were <0.5, suggesting that multicollinearity did not affect the findings. As certain states and certain categories of respondents were oversampled in the survey, sample weights were used to restore the representativeness of the sample (IIPS & Macro International, 2007). Analyses were conducted using the SPSS statistical software package version 19 (IBM SPSS Statistics, Chicago, IL, USA).

### Ethical considerations

The NFHS-3 received ethical approval from the International Institute for Population Science's Ethical Review Board and the Indian Government. Participation in the survey was totally voluntary. The survey obtained written informed consent from each respondent (men and women) before asking questions, and separately before obtaining height and weight measurements and before blood collection for hemoglobin measurement. The analysis presented in this study is thus based on secondary analysis of existing survey data with all identifying information removed.

## Results

### Profile of the respondents and prevalence of symptoms of preeclampsia/eclampsia

Table1 shows the distribution of the respondents by selected characteristics as well as the reported prevalence of preeclampsia/eclampsia in pregnancy by predictor variables. More than three-fourth of the sample women (78%) live in households using biomass and solid fuels (wood, dung cakes, crop residues or coal/coke/lignite or charcoal) and one of five (22%) live in households using cleaner fuels (kerosene, liquid petroleum gas, biogas, or electricity). Among other sample characteristics, one of four belonged to parity 1, one of five women (19%) reported ever termination of pregnancy, while 1% had a twin pregnancy. Two of five women were mildly anemic, 15% were obese, 1.5% current smokers, 2.3% current drinkers, 1.2% diabetic, and 1.2% asthmatic. Three-fourths of the women were in young age category (age 15–29), almost half the women had no education, the majority were Hindus, two-fifth belonged to ‘other backward class’, and two of five women belonged to households in the lowest wealth quintile. The unadjusted prevalence of symptoms suggestive of preeclampsia/eclampsia was found significantly higher (1.4%; *P *<* *0.0001) among women residing in households using biomass and solid fuel and in the other following groups: parity greater than four (1.7%); twin pregnancy (3.1%); previously terminated pregnancy (1.4%); mild to moderate anemia (1.3–1.5%); underweight and normal weight (1.3%); current smokers (1.7%); current alcohol drinkers (2.6%); diabetics (1.6%); asthmatics (4.7%); no education (1.6%); other religion (2.5%); Scheduled Tribes (2.2%); households belonging to lowest wealth quintile (2.0%); residence in rural areas (1.4%) and residence in central part of India (1.7%).

### Effects of cooking smoke on preeclampsia/eclampsia

Table2 shows the estimated effects of household cooking fuel, and selected maternal, health and lifestyle factors and demographic and socioeconomic characteristics on the likelihood of preeclampsia/eclampsia symptoms among women in alternative models. Model 1 in Table2 shows that unadjusted odds of reporting preeclampsia/eclampsia symptoms were more than three times higher (OR: 3.35; 95% CI: 2.36–4.76; *P* < 0.0001) among women living in households using biomass and solid fuels for cooking than among those living in households using cleaner fuels for cooking. Even when the seven socioeconomic and demographic variables, three maternal factors, and six health- and lifestyle-related factors are included in Model 2, cooking with biomass and solid fuels still has a large and statistically significant effect (OR: 2.21; 95% CI: 1.26–3.87; *P* = 0.003) on the likelihood of preeclampsia/eclampsia symptoms among women.

**Table 2:** Unadjusted and adjusted effects (OR, 95% CI) of indoor air pollution from biomass and solid fuel combustion on the likelihood of preeclampsia/eclampsia during pregnancy among women age 15–49 years who had a live birth in the 5 years preceding the survey, India, 2005–2006

Characteristics	Preeclampsia/Eclampsia			
	Model 1		Model 2	
	OR	95% CI	OR	95% CI
Household cooking fuel use				
Clean fuel (ref)	–		–	–
Biomass and solid fuel	3.35	2.36–4.76	2.21	1.26–3.87
Maternal factors				
Total children ever born				
1 (ref)	–		–	
2–3	1.24	0.96–1.61	1.19	0.86–1.63
4+	2.10	1.62–2.72	1.29	0.87–1.92
Type of pregnancy				
Singleton (ref)	–		–	
Twin	2.65	1.43–4.92	3.08	1.64–5.80
Ever had a terminated pregnancy				
No (ref)	–		–	
Yes	1.34	1.08–1.67	1.44	1.14–1.80
Health and lifestyle factors				
Body mass index				
Underweight (≤18.5 kg/m^2^)	1.01	0.83–1.23	0.92	0.75–1.13
Normal (18.5–22.9 kg/m^2^) (ref)	–		–	
Overweight (23.0–24.9 kg/m^2^)	0.65	0.42–1.01	0.96	0.61–1.50
Obese (≥25.0 kg/m^2^)	0.41	0.24–0.69	0.86	0.36–1.22
Current tobacco smoking				
No (ref)	–		–	
Yes	1.50	0.81–2.79	0.97	0.51–1.83
Drinks Alcohol				
No (ref)	–		–	
Yes	2.34	1.54–3.57	1.29	0.62–2.31
Diabetes				
No (ref)	–		–	
Yes	1.34	0.66–2.72	0.87	0.39–1.92
Asthma				
No (ref)	–		–	
Yes	4.33	2.79–6.73	4.67	2.53–8.62
Anemia level				
Not anemic (ref)	–		–	
Mild	1.54	1.23–1.92	1.40	1.11–1.77
Moderate	1.78	1.37–2.31	1.52	1.16–2.00
Severe	1.06	0.47–2.42	0.89	0.39–2.03
Background factors				
Age				
15–29 (ref)	–		–	
30–39	1.35	1.10–1.66	1.04	0.74–1.40
40–49	1.25	0.73–2.16	0.82	0.46–1.45
Education				
No education (ref)	–		–	
Primary	0.75	0.58–0.99	1.03	0.72–1.46
Secondary	0.45	0.35–0.57	0.91	0.64–1.29
Higher	0.14	0.06–0.33	0.35	0.13–1.00
Religion				
Hindu (ref)	–		–	
Muslim	0.92	0.71–1.20	0.96	0.70–1.32
Christian	0.64	0.28–1.43	0.68	0.25–1.84
Sikhs	0.68	0.25–1.81	0.98	0.35–2.77
Others	2.21	1.30–3.77	1.81	0.99–3.30
Caste/tribe				
Scheduled caste (ref)	–		–	
Scheduled tribes	1.81	1.35–2.43	1.66	1.09–2.53
Other backward class	0.87	0.68–1.12	1.14	0.81–1.61
General	0.65	0.48–0.86	1.01	0.67–1.51
Missing caste	0.80	0.43–1.51	0.99	0.42–2.31
Wealth index				
Lowest (ref)	–		–	
Second	0.64	0.51–0.82	0.73	0.52–1.02
Middle	0.50	0.38–0.65	0.70	0.48–1.03
Fourth	0.33	0.24–0.45	0.72	0.46–1.14
Highest	0.28	0.20–0.41	1.12	0.52–1.90
Place of residence				
Urban (ref)	–		–	
Rural	2.24	1.72–2.91	1.16	0.79–1.71
Geographic regions				
North (ref)	–		–	
Northeast	1.31	0.79–2.18	0.92	0.46–1.83
Central	1.66	1.22–2.26	1.35	0.92–1.98
East	1.34	0.97–1.85	0.78	0.51–1.20
West	0.69	0.45–1.06	0.56	0.33–0.95
South	0.31	0.19–0.51	0.25	0.14–0.47
Total	34,204		30,745	

(ref) denotes reference category.

For variable definition see Table1.

### Effects of the confounders on preeclampsia/eclampsia

The discussion of the adjusted effects of the covariates/confounders focuses on the full model (Model 2) in Table2. With other variables controlled, self-reported asthma (OR = 4.67; 95%: 2.53–8.62; *P* < 0.001), mild (OR = 1.40; 95%: 1.11–1.77; *P* = 0.004) and moderate (OR = 1.34; 95%: 1.02–1.76; *P* = 0.002) anemia, twin pregnancy (OR = 3.08; 95%: 1.64–5.80; *P* < 0.0001), and terminated pregnancy (OR = 1.44; 95%: 1.14–1.80; *P* = 0.002) were associated with a higher likelihood of women reporting preeclampsia/eclampsia symptoms. Women belonging to a scheduled tribe (OR = 1.66; 95%: 1.09–2.53; *P* = 0.002) also had a higher likelihood of reporting preeclampsia/eclampsia symptoms. Women with higher educational attainment (OR: 0.35; 95% CI: 0.13–1.00), and women residing in the western (OR: 0.56; 95% CI: 0.33–0.95) and southern region (OR: 0.25; 95% CI: 0.14–0.47) of India had a lower likelihood of reporting preeclampsia/eclampsia symptoms. The adjusted likelihood of women reporting preeclampsia/eclampsia symptoms did not vary significantly by urban/rural residence, wealth status of the household, religion, age of the women, and the presence of diabetes, alcohol drinking, tobacco smoking, BMI, and parity.

## Discussion

To our knowledge, our study is the first to empirically estimate the associations of indoor air pollution from biomass and solid fuel combustion and reported symptoms suggestive of preeclampsia/eclampsia in a large nationally representative sample of Indian women. We observed an increased risk of preeclampsia/eclampsia symptoms among women using biomass and solid fuel for cooking and the association remained significant when we adjusted for several maternal, health- and lifestyle-related factors and socio-demographic characteristics.

### Result in context of other studies

Recent studies that have linked ambient air pollution (mainly outdoor) to gestational hypertension, and preeclampsia were primarily based on developed countries (van den Hooven et al., 2009, 2011; Rudra et al., 2011; Lee et al., 2012; Pereira et al., 2013; Vinikoor-Imler et al., 2012; Wu et al., 2009, 2011), and there is limited empirical evidence of this association or association with indoor air pollution from household inefficient cooking fuel use and preeclampsia/eclampsia in India and other developing countries. Increased risk of gestational hypertension and preeclampsia among the mothers exposed to air pollution may explain the relationships between air pollution and adverse birth outcomes such as fetal growth restriction, low birth weight rate and preterm birth (Yurdakök, 2013). A recent US study found an association between first trimester PM10 and O3 air pollution exposures and increased blood pressure in the later stages of pregnancy (Lee et al., 2012). Maternal PM10 exposure was associated with an increased risk of pregnancy-induced hypertension (OR: 1.72; 95% CI, 1.12–2.63, per 10-*μ*g/m^3^ increase) in 7006 women participating in a prospective cohort study in the Netherlands (van den Hooven et al., 2011). Another epidemiologic study showed that exposure to local traffic-generated air pollution during pregnancy increases the risk of preeclampsia and preterm birth (Pereira et al., 2013). In this study, the risk of preeclampsia increased 33% (OR: 1.33, 95% CI, 1.18–1.49) and 42% (OR: 1.42, 95% CI, 1.26–1.59) for the highest nitric oxides (NOx) and PM2.5 exposure quartiles, respectively.

### Mechanisms

The biological mechanisms of how air pollution leads to adverse pregnancy health and the exact etiologic mechanism discerning the effect of indoor air pollution on preeclampsia/eclampsia occurrence are not yet well understood partially because the condition is a multisystem disorder of unknown etiology (Sibai, 1998). However, it is known that particulate matter air pollution is capable of augmenting the development and progression of atherosclerosis and may potentially contribute to hypertension (Brook et al., 2010). Preeclampsia and vascular atherosclerosis may share common pathways in relation to pollutants (Duckitt and Harrington, 2005; Kaaja and Greer, 2005). Ambient air pollution has been directly correlated with endothelial dysfunction (Törnqvist et al., 2007), a precursor associated with preeclampsia (Steegers et al., 2010). It is suspected that emissions may also contribute to an anti-angiogenic state (Ejaz et al., 2009) that may in turn contribute to the development of preeclampsia (Young et al., 2010).

The other biological plausibility of air pollution (specifically fine particulate matter) affecting health has been discussed by Pope and Dockery (2006), focusing on exposure effects on blood pressure (Brook, 2008), aspects of substantial relevance to health during pregnancy (Yoder et al., 2009). There is increasing evidence that ambient air pollution may act via systemic inflammation and increased blood pressure (Brook, 2008; Brook and Rajagopalan, 2009; Lee et al., 2012), increasing the risk of cardiovascular mortality, adverse pregnancy outcomes (Rückerl et al., 2011; Stieb et al., 2012; Sun et al., 2010), and adverse birth outcomes (Bobak, 2000; Liu et al., 2003; Malmqvist et al., 2011; Slama et al., 2009; Wilhelm and Ritz, 2003). Other experimental and observational evidence also equally indicates that exposure to ambient air pollution, particularly ultrafine particles, induces oxidative stress and consequently inflammation (Redman and Sargent, 2003; Sibai et al., 2005; Terzano et al., 2010). Through systemic inflammation, some particles penetrate deeper into the lung are able to interact with immune cells and even exhibit systemic effects entering the bloodstream (Yurdakök, 2013).

### Implications of indoor air pollution for prevention of pregnancy complications in India

The findings from this study suggests that there is an urgent need for public health actions in India, including more vigorous information campaigns designed to inform people about the risks of exposure to cooking smoke and programs to promote improved cooking stoves designed to reduce exposure to smoke by means of improved combustion and increased ventilation (Mishra, 2003) which could in turn thus minimize the adverse health effect including preeclampsia/eclampsia. Interventions that reduce the burden of biomass smoke exposure also are urgently required in India (Salvi and Barnes, 2010). Feasible low-cost measures that could reduce the devastating health outcomes of exposure to indoor biomass smoke include cooking outdoors, cooking for shorter periods, improving ventilation by adding more windows around cooking areas or by building chimneys above stoves, improving stove construction and technology, or encouraging the use of cleaner or energy-efficient fuels such as LPG, ethanol, or biogas (Salvi and Barnes, 2009). Substitution of traditional open fires with locally produced improved stoves has been shown to have significant health benefits (Chapman et al., 2005; Romieu et al., 2009; Smith-Sivertsen et al., 2009). To make community development programs effective, local needs and community participation should be given high priority (Mishra, 2003). Also, programs to reduce exposure to cooking smoke during pregnancy should be promoted, in addition to strengthening asthma prevention and treatment programs. Further, it is also important to increase awareness about the adverse health effects of solid fuel smoke inhalation, particularly during pregnancy, among physicians and health administrators as well (Salvi and Barnes, 2010), which may improve the diagnosis and treatment of affected patients and trigger preventive actions through education, research, and policy change. A focus on community education highlighting the harmful effect of biomass fuel smoke exposure may mobilize demand for improved stove installations and better household ventilation. This may also lead to the further development of community development programs and, specifically, strategies for poverty reduction, which are currently underway in India.

### Implications of preeclampsia/eclampsia for prevention of pregnancy complications in India

India is in the midst of a demographic, epidemiological and nutrition transition characterized by a growing population, increasing urbanization, a shift in the patterns of diseases and changes in lifestyle (Shetty, 2002). The past decade has seen a dramatic increase in lifestyle-related non-communicable diseases including obesity, diabetes mellitus, high blood pressure, coronary heart disease, stroke, and cancers (WHO, 2003b). Given that preeclampsia shares many risk factors with cardiovascular disease (obesity, type-2 diabetes, high blood pressure among others), it is expected that the increase in cardiovascular disease risk factors in women of childbearing age could translate into a higher incidence of preeclampsia and its complications (e.g. eclampsia), thus increasing the risk to pregnant women in India. Ten percent of women have high blood pressure during pregnancy, and eclampsia and preeclampsia complicates 2–8% of pregnancies (Duley, 1992, 2009). The Millennium Development Goals have placed maternal health at the core of the struggle against poverty and inequality, as a matter of human rights. Increasing awareness of maternal mortality as a public health priority, for both maternal and child health, has been important and has helped implementation of improved health services in developing countries. Millennium Development Goal 5 calls for a reduction by three-quarters, between 1990 and 2015, in the maternal mortality ratio (www.un.org/millenniumsgoals). Thus, with the target of the Millennium Development Goals in sight, preeclampsia/eclampsia needs to be identified as a priority area in reducing maternal mortality and morbidity in developing countries including India.

### Strengths and weaknesses of the study

The main strength of the present study is the large nationally representative dataset from the National Family Health Survey, which allowed us to examine the effect of indoor air pollution from biomass and solid fuel combustion along with a range of socioeconomic, maternal, and health and lifestyle factors in relation to preeclampsia/eclampsia risk in the Indian women of childbearing age. A particular strength was that we had individual-level information on socioeconomic status which allowed for further investigations of the exposures and the outcomes of interest. Further, the large sample size provided adequate power to identify various risk factors and compensated for the large ethnic variations in Indian populations. Additionally, the survey was conducted using an interviewer-administered questionnaire in the native language of the respondent using local, commonly understood terms for pregnancy-related health problems. A total of 18 languages were used with back translation to English to ensure accuracy and comparability.

However, due to the general challenges of measuring hypertensive disorders in population-based studies, the measurement of symptoms of suggestive of preeclampsia/eclampsia in the NFHS also has apparent limitations. Case ascertainment was based on self-reported symptoms as experienced by women during their last pregnancy rather than a clinical assessment: health problems during pregnancy such as difficulty with vision during daylight, swelling of the legs, body, or face and seizures/convulsions during pregnancy were used as proxy indicators of preeclampsia/eclampsia. These self-reports may be subjected to recall bias leading to the over-reporting of major symptoms among those affected. Also self-reports, especially in rural areas, can be flawed owing to several factors such as lack of awareness, low educational status and hesitation to disclose the problem. Therefore, although we cannot exclude misclassification within this context, it is unlikely that severe preeclampsia/eclampsia symptoms were missed. It was also not possible to identify the gestational onset of preeclampsia/eclampsia from the given data.

Limitations also include the cross-sectional study design, which thereby limits conclusions regarding causality. In cross-sectional data, there may be errors in the measurement of the variables, some relevant variables may be omitted or unobservable, ‘dependent’ and ‘independent’ variables may in fact be determined simultaneously, the sample on which the estimation is based may be biased, independent variables may be correlated (‘multicolinearity’), the error term may not have a constant variance, and outliers may have a strong influence on the results (Angrist et al., 1996). But we assessed the possibility of multicollinearity between the covariates where all pairwise Pearson's correlation coefficients were <0.5, suggesting that multicollinearity did not affect the findings. In cross-sectional data, there may occur simultaneity, or reverse causality, when the independent variable of interest and the outcome are determined jointly, or at the same time, making the direction of causality unclear. Also, there may be problem of omitted variable bias as the confounders are typically self-selected. As Angrist and Alan (2001) pointed out, it is rare that a theory specifies all of the variables that must be controlled for in a given relationship and even if it did, it would be nearly impossible to observe and measure them all. While rich data sets with extensive background characteristics are helpful in this regard, omitted variable bias remains problematic in multivariate regression analysis. The idea of self-selection is that the confounders and covariates were selected by the researcher based on the previous knowledge and their association found in earlier studies. The basis for these decisions may be related to characteristics that we can observe, but they may also be based on characteristics that we cannot observe—a problem refer to as ‘unobserved heterogeneity’. So the results in this cross-sectional analysis should be interpreted with caution as there may be cases of residual confouding.

Nevertheless, in India, where clinical data on preeclampsia/eclampsia are mostly unavailable or available only for a specific population or at hospital admission, this analysis of preeclampsia/eclampsia in a nationally representative sample of adult women provides important evidence of the effect of biomass and solid fuel exposure on preeclampsia/eclampsia risk. However, this is an observational finding and uncontrolled confounding cannot be excluded as an explanation for the association. Well-designed epidemiologic studies with better smoke exposure assessment, personal exposure, ambient concentration estimates and clinical measures of preeclampsia/eclampsia is needed in a developing country setting to validate these findings and to better understand the etiology of preeclampsia/eclampsia in Indian mothers.

## Conclusions

We observed an increased prevalence of self-reported symptoms suggestive of preeclampsia/eclampsia with exposure to indoor air pollution from biomass and solid fuel combustion in a large nationally representative sample of adult Indian women. These findings have important program and policy implications for countries such as India, where large proportions of the population rely on polluting biomass fuels for cooking and space heating and have an additional burden of high maternal and infant mortality and morbidity. Indoor air pollution is a major public health threat in India which requires greatly increased efforts in the areas of more intensive research and policy-making. Research on its health effects such as preeclampsia/eclampsia should be put forth and strengthened in addition to other health effects. A more systematic approach to the development and evaluation of interventions is required, with clearer recognition of the interrelationships between poverty and dependence on polluting fuels and exposure of vulnerable population.

## Supporting Information

**Table S1.**Descriptive statistics (Case Processing Summary) of each the variables studied with missing cases. **Data S1.** Advantage and disadvantage of using the wealth index and items used to compute wealth index in NFHS-3.
