# Supplementary material for: Effect of Indoor air pollution from biomass and solid fuel combustion on symptoms of preeclampsia/eclampsia in Indian women
Source: Indoor Air. 2014 Aug 11;25(3):341–52. doi: 10.1111/ina.12144 (PMC4431462; doi:10.1111/ina.12144)
Supplement: Supplementary file 1 — Table S1.Descriptive statistics (Case Processing Summary) of each the variables studied with missing cases. Data S1. Advantage and disadvantage of using the wealth index and items used to compute wealth index in NFHS-3. [file ina0025-0341-sd1.docx]

**10.1111/ina.12144**

**Online supporting information for the following article published in Indoor Air**

**DOI:** 10.1111/ina.12144

**Effect of Indoor Air Pollution from Biomass and Solid fuel Combustion on Symptoms of Preeclampsia/Eclampsia in Indian Women**

*Running Head: Indoor air pollution and Preeclampsia/Eclampsia*

**Author(s): Sutapa Agrawal1*, Shelby Yamamoto2**

1 South Asia Network for Chronic Disease, Public Health Foundation of India, New Delhi, INDIA

2 Department of Non-communicable Disease Epidemiology, London School of Hygiene and Tropical Medicine, London, UK

*Address for communication and reprint request: Sutapa Agrawal, Epidemiologist, South Asia Network for Chronic Disease, Public Health Foundation of India, Plot no 47, Sector 44, Gurgaon, Haryana-122002, India, Tel: 0124- 4781400 Ext 4488; Mob:+919650155334; E-mail: [sutapaiips@rediffmail.com](mailto:sutapaiips@rediffmail.com  ) or [sutapa.agrawal@phfi.org](mailto:sutapa.agrawal@phfi.org); Website:www.sancd.org/www.phfi.org

**Data S1: Advantage and disadvantage of using the wealth index and items used to compute wealth index in NFHS-3** Wealth index in the NFHS-3 is an index of the economic status of households and has been developed and tested in a large number of countries in relation to inequalities in household income, use of health services, and health outcomes (Rutstein et al., 2000). It is an indicator of the level of wealth that is consistent with expenditure and income measures (Rutstein, 1999). The economic index was constructed using household asset data and housing characteristics. It is also easy to obtain information from households on economic proxies through simple questions or direct observation (Sahn and Stifel, 2003) and these indicators have a direct influence on health (Howe et al., 2008). The NFHS-3 wealth index is based on the following 33 assets and housing characteristics: Household electrification; type of windows; drinking water source; type of toilet facility; type of flooring; material of exterior walls; type of roofing; cooking fuel; house ownership; number of household members per sleeping room; ownership of a bank or post-office account; and ownership of a mattress, a pressure cooker, a chair, a cot/bed, a table, an electric fan, a radio/transistor, a black and white television, a colour television, a sewing machine, a mobile telephone, any other telephone, a computer, a refrigerator, a watch or clock, a bicycle, a motorcycle or scooter, an animal-drawn cart, a car, a water pump, a thresher, and a tractor.

The wealth index, as a proxy of consumption expenditure, is a subject of intense debate and discussion, though its utility in predicting differentials in health outcome and health care utilisation has been established. A number of studies demonstrate that the wealth index is a good proxy of economic status (Filmer and Pritchett, 2001; Wagstaff and Watanabe, 2003; Rutstein and Johnson, 2004). Other studies, however, also describe the wealth index as a weak predictor of consumption expenditure and a poor measure of inequality (Montgomery et al., 2000; Lindleow, 2006; Howe et al., 2008). The Spearman’s rank correlation of wealth index and consumption expenditure varies largely across countries; from 0.37 in Mozambique (Sahn and Stifel, 2003) to 0.56 in Indonesia and 0.64 in Nepal (Filmer and Pritchett, 2001). Besides proxies for a direct economic measure, the wealth index tends to have an urban bias, does not segregate the poor, does not identify the poorest of the poor and it is sensitive to the choice of indicators included in its calculation (Rutstein, 2008; Houweling et al., 2003). Howeling et al. (2003), using the DHS data from 10 developing countries, have found that the magnitude of health inequalities is sensitive to the choice of asset items included in the index. Bollen et al. (2002) have found that the choice of proxies makes a difference in the economic status of the household.

**References**

Bollen KA, Glanville JL, Stecklov G. (2002) Economic status proxies in studies of fertility in developing countries: Does the measure matter? Population Studies, 56(1), 81-96.

Filmer D, Pritchett LH (2001) Estimating wealth effects without expenditure data- or tears: An application to educational enrollments in states of India, Demography, 38(1), 115-132.

Houweling TAJ, Kunst AE, Mackenbach JP (2003) Measuring health inequality among children in developing countries: does the choice of indicator of economic status matter? International Journal for Equity in Health, 2, 8-19.

Howe DL, Hargreaves JR, Huttly SRA (2008) Issues in the construction of wealth indices for the measurement of socio-economic position in low- income countries, Emerging Themes in Epidemiology, 5(3). <http://www.ete-online.com/content/5/1/3>

Lindleow M (2006) Sometimes more equal than others: how health inequalities depend on the choice of welfare indicator, Health Economics, 15(3), 263-279.

Montgomery MR, Gragnolati M, Burke KA, Paredes E (2000) Measuring living standards with proxy variables Demography, 27, 155-174.

Rut Stein SO, Johnson K (2004) The DHS Wealth Index, DHS Comparative Reports N. 6, ORC Macro, Maryland.

Sahn D, Stifel D (2003) Exploring alternative measures of welfare in the absence of expenditure data Review of Income and Wealth, 49(4), 463-489.

Rutstein S, Johnson K, Gwatkin D (2000) Poverty, health inequality, and its health and demographic effects. Paper presented at the 2000 Annual Meeting of the Population Association of America, Los Angeles, California.

Rutstein S (1999) Wealth versus expenditure: Comparison between the DHS wealth index and household expenditures in four departments of Guatemala. Calverton, Maryland: ORC Macro.

Rutstein SO (2008) Further Evidence of the Effects of Preceding Birth Intervals on Neonatal, Infant, and Under-Five-Years Mortality and Nutritional Status in Developing Countries: Evidence from the Demographic and Health Surveys. DHS Working Papers No. 41.

Wagstaff A, Watanabe N (2003) What difference does the choice of SES make in health inequality measurement? Health Econ, 12(10):885-90.

Table S.1: Descriptive statistics (Case Processing Summary) of each the variables studied with missing cases.

|  | | | | | | |
| --- | --- | --- | --- | --- | --- | --- |
|  | **Cases** | | | | | |
|  | **Valid** | | **Missing** | | **Total** | |
|  | **N** | **Percent** | **N** | **Percent** | **N** | **Percent** |
| Household cooking fuel use | 36127 | 91.2 | 3484.698 | 8.8 | 39611.698 | 100.0 |
| Parity | 39611^a^ | 100.0 | .698 | .0 | 39611.698 | 100.0 |
| Type of pregnancy | 39612^a^ | 100.0 | 0 | .0 | 39611.698 | 100.0 |
| Ever had a terminated pregnancy | 39611^a^ | 100.0 | .698 | .0 | 39611.698 | 100.0 |
| Body Mass Index | 38003^a^ | 95.9 | 1608.698 | 4.1 | 39611.698 | 100.0 |
| Current tobacco smoking | 39612^a^ | 100.0 | 0 | .0 | 39611.698 | 100.0 |
| Drinks alcohol | 39601^a^ | 100.0 | 10.698 | .0 | 39611.698 | 100.0 |
| Diabetes | 39592^a^ | 100.0 | 19.698 | .0 | 39611.698 | 100.0 |
| Asthma | 39588^a^ | 99.9 | 23.698 | .1 | 39611.698 | 100.0 |
| Anemia level | 37249^a^ | 94.0 | 2362.698 | 6.0 | 39611.698 | 100.0 |
| Age | 39612^a^ | 100.0 | 0 | .0 | 39611.698 | 100.0 |
| Education | 39610^a^ | 100.0 | 1.698 | .0 | 39611.698 | 100.0 |
| Religion | 39610^a^ | 100.0 | 1.698 | .0 | 39611.698 | 100.0 |
| Caste/tribe | 39454^a^ | 99.6 | 157.698 | .4 | 39611.698 | 100.0 |
| Wealth index | 39611^a^ | 100.0 | .698 | .0 | 39611.698 | 100.0 |
| Place of residence | 39612^a^ | 100.0 | 0 | .0 | 39611.698 | 100.0 |
| Geographic Regions | 39611^a^ | 100.0 | .698 | .0 | 39611.698 | 100.0 |
| ^a^ Number of valid cases is different from the total count in the cross tabulation table because the cell counts have been rounded. | | | | | | |
